# Parental Values and Children’s Resilience: The Role of Socio-Demographic Factors in a Romanian Sample

**DOI:** 10.3390/bs16091623

**Published:** 2026-09-10

**Authors:** Lucica-Emilia Coșa, Irina-Mihaela Trifan

**Affiliations:** 1 Department of Teacher Training, George Emil Palade University of Medicine, Pharmacy, Science, and Technology of Targu Mures, 540139 Târgu Mureș, Romania; lucica.cosa@umfst.ro; 2 Department of Sciences and Letters 2, Faculty of Sciences and Letters “Petru Maior”, George Emil Palade University of Medicine, Pharmacy, Science, and Technology of Targu Mures, 540139 Târgu Mureș, Romania

**Keywords:** resilience, children, parental values, Schwartz values theory, CYRM-28, Romanian cultural context

## Abstract

**Background:** Children’s resilience is a dynamic, multisystemic process shaped by cultural context, supportive relationships, and family values. In Romania, research on children’s resilience and the role of parental values remains limited. **Objective:** This study examined the relationship between children’s resilience, socio-demographic factors (parental educational level, economic status, family structure, religious affiliation, and ethnicity), and parental values based on Schwartz’s value model. We hypothesized that socially oriented values (security, conformity, tradition, universalism, and benevolence) would be associated with higher resilience. **Method:** A cross-sectional correlational study was conducted with 245 children aged 8–15 years (*M* = 10.58, *SD* = 1.95) and one parent per child. Children’s resilience was assessed using the Child and Youth Resilience Measure (CYRM-28), parental values using the Short Schwartz’s Value Survey (SSVS), and socio-demographic data through a parental questionnaire. **Results:** Higher resilience was associated with higher parental educational level and with parental values reflecting conservation and self-transcendence. In the regression model, parental educational level, conservation values, Orthodox religious affiliation, and intact family structure were independently associated with resilience and jointly accounted for 22.6% of the variance. Younger children had higher resilience, but age was not a significant predictor in regression analysis. **Conclusions:** The findings support the socio-ecological perspective on resilience and provide among the first empirical evidence from Romania that parental values, together with family and cultural resources, are associated with children’s resilience.

## 1. Introduction

Resilience in children and adolescents represents a central construct in contemporary developmental psychology, with important implications for mental health, academic achievement, and long-term life outcomes (Masten, 2018). Defined as the dynamic capacity of a system to adapt successfully to adversity, resilience is now understood not as a stable individual trait but as a multidimensional process shaped by biological, psychological, familial, social, and cultural resources (Ungar & Liebenberg, 2011). This process-oriented perspective, reinforced by Ungar’s cross-cultural research, has gradually shifted the understanding of resilience away from the traditional “heroic” perspective toward an ecological approach that views children as embedded within multiple interacting systems of support (Ungar & Liebenberg, 2011).

The present study builds upon two complementary lines of research: the role of parental values and socioeconomic factors as determinants of children’s resilience, and the contribution of cultural and parental values to fostering resilience. Previous research provides consistent evidence of the relationship between family value systems and resilience. In this regard, Sagone and De Caroli (2016), in a sample of 307 Sicilian adolescents aged 14 to 18 years, demonstrated that self-transcendence values were positively associated with all dimensions of resilience, whereas conservation values were related to adaptability, self-control, and commitment. Likewise, Collins et al. (2024), in a study involving 738 primary school children aged 6 to 12 years, found that values oriented toward healthy development were associated with higher levels of subjective well-being. This relationship was stronger among girls and children aged 9 to 12 years and was partially mediated by perceived social support. Furthermore, Nguyen et al. (2016), in a study conducted with 845 university students from both Eastern and Western cultural contexts, identified a mediated pathway linking parental storytelling, the transmission of values, and resilience.

Despite these findings, empirical evidence regarding children’s resilience in Romania remains limited. The available literature is based primarily on studies conducted in Western cultural contexts or educational systems that differ substantially from the Romanian context, whereas samples from Eastern Europe remain underrepresented. This gap is particularly relevant because Romania’s sociocultural characteristics, such as strong family bonds, religious traditions, and community values, may constitute important protective resources that support children’s resilience. From an ecological perspective, however, the ways in which these resources contribute to children’s resilience have not yet been systematically investigated.

### 1.1. Defining and Conceptualizing Resilience

The systematic study of resilience has its roots in the pioneering research initiated in the 1970s by Garmezy, Murphy, Rutter, and Werner. The history of the field has been comprehensively documented by Cicchetti and Garmezy (1993), who trace the origins of systematic resilience research to Garmezy’s Project Competence (Garmezy, 1971, as cited in Cicchetti & Garmezy, 1993) and Werner and Smith’s Kauai Longitudinal Study (Werner & Smith, 1982, as cited in Cicchetti & Garmezy, 1993; see also Werner, 1989, 1993).

Definitions of resilience have evolved through four conceptual waves, as described by Masten and Wright (2010, as cited in Pooley & Cohen, 2010): the first focused on defining the concept; the second on identifying associated variables; the third on testing intervention strategies; and the fourth on achieving multilevel and multidisciplinary integration (Pooley & Cohen, 2010). The most influential definition remains that proposed by Masten et al., who defined resilience as “the process of, capacity for, or outcome of successful adaptation despite challenging or threatening circumstances” (Masten et al., 1990, p. 426). The same work identifies three manifestations of resilience: (a) positive outcomes among high-risk children; (b) sustained competence under conditions of stress; and (c) recovery following trauma. The authors concluded that human psychological development is supported by powerful protective mechanisms and that the long-term consequences of adversity generally emerge only when organic systems are compromised or when the normative protective processes within caregiving systems are severely disrupted (Masten et al., 1990).

The contemporary perspective has been further advanced by Masten, who defines resilience as the capacity of a dynamic system to adapt successfully, through processes operating across multiple levels, to challenges that threaten its functioning, survival, or development (Masten, 2018). This definition applies across multiple levels of analysis, including the individual, family, community, and ecosystem. Southwick et al. (2014) support this conceptualization in their interdisciplinary synthesis of resilience research.

The expression *ordinary magic* was originally introduced by Masten (2001), who argued that one of the most remarkable findings emerging from research on children exposed to adversity is precisely the ordinary nature of resilience: it arises from common rather than exceptional adaptive processes. This conclusion was further elaborated by Masten and Obradović (2008), who argued that resilience is grounded in fundamental adaptive systems shaped throughout biological and cultural evolution. These systems include the neurobiological mechanisms involved in stress regulation, attachment relationships, adaptive thinking and problem-solving, executive functioning and self-regulation, coping processes, and reward systems. From this perspective, interventions designed to strengthen resilience in children and families should focus on preventing trauma, reducing stress and vulnerability, increasing protective resources, mobilizing core adaptive systems, and promoting positive developmental cascades across multiple ecological levels and generations (Masten, 2019).

Complementing this perspective, Rutter’s definition, cited by Southwick et al. (2014) and supported by the review of his 2006 book (Leckman, 2007), emphasizes the importance of evidence-based inference. According to this view, resilience is an inference that some individuals achieve better outcomes than others despite experiencing comparable levels of adversity (Leckman, 2007; Southwick et al., 2014). Rutter systematically documented the contributions of both genetic and environmental factors to resilience, moving beyond the traditional diathesis-stress model through the concept of differential susceptibility. This concept suggests that the same individuals who are genetically more vulnerable to adverse experiences are also those who benefit the most from supportive environments (Belsky & Pluess, 2009; Cicchetti & Rogosch, 2012).

### 1.2. The Cultural Dimension of Resilience

The shift from an individual perspective to an ecological and multicultural perspective has generated an important line of research in the field of resilience. International studies led by Ungar have shown that resilience is shaped by both universal factors and cultural and contextual characteristics, and that its expression varies according to the environments in which children grow and develop (Ungar, 2008; Ungar et al., 2008).

Clauss-Ehlers describes a culturally centered model of resilience as a dynamic and interactive process in which individuals cope with stress through the interplay of their personal characteristics, cultural values, contextual influences, and facilitating factors within the sociocultural environment (Clauss-Ehlers, 2008, as cited in Johns et al., 2014). Likewise, Marks et al. (2020) emphasize the need for ecological, intersectional, and culturally responsive approaches to advance clinical research on health disparities between minority and majority populations. The cultural dimension of resilience was most explicitly addressed by Panter-Brick in her contribution to Southwick et al. (2014), based on systematic face-to-face interviews with more than one thousand Afghan families. She concluded that, if resilience were to be reduced to a single word in the Afghan context, that word would be *hope*. What distinguished more resilient families was their ability to maintain a sense of hope that gave meaning to suffering and provided a coherent narrative linking the past, present, and future (Southwick et al., 2014).

Recent empirical evidence has quantified these cultural differences. Skevington and the WHOQOL SRPB Group (N = 3019 participants from 15 countries) demonstrated, using a sequential multiple linear regression model, that the final model explained 52% of the variance in resilience, with quality of life accounting for 37% of the explained variance and culture contributing an additional 12% (*p* < 0.0001) (Skevington & The WHOQOL SRPB Group, 2020). The study further showed that the spiritual dimensions of quality of life—including meaning in life, awe, wholeness and integration, kindness to others, and hope—were the strongest predictors of culturally embedded resilience. Blessin et al. (2022), in a comparative meta-analysis of 175 Western and 46 Eastern studies examining resilience-building interventions, reported that interventions implemented in Eastern countries produced significantly larger effects on resilience (SMD = 0.48, 95% CI [0.28, 0.67], *p* < 0.0001; 43 studies involving 6248 participants). These interventions were more frequently delivered in group settings and were more likely to involve family caregivers. Complementing these findings, the network meta-analysis conducted by Ciren et al. (2026), based on 46 randomized controlled trials (N = 8729; mean age = 15.6 years), found that physical activity, psychotherapy, mindfulness, and skills training significantly improved resilience compared with control conditions. The same context dependence applies to the contextual variables examined in the present study. Religion and spirituality may support resilience through optimism, meaning-making, coping and social support, but the reported effects are small and vary with age, ethnic background and the way religiosity is measured (Yonker et al., 2012). The association between family structure and children’s outcomes likewise differs from one country to another (Fismen et al., 2022), and findings on ethnic background are inconsistent: some studies report better well-being among children with an immigrant background than among their native peers, others similar or less favourable outcomes (Tangelder et al., 2025).

### 1.3. Protective Factors and the Role of Parents

A comprehensive synthesis of research on protective factors is provided by Masten (2018, 2019), who argues that these factors include effective caregiving and other supportive relationships, problem-solving and self-regulation skills, self-efficacy, optimism, and a sense of meaning in life. Self-efficacy in particular has also emerged as a robust predictor of related developmental outcomes within the Romanian context: Coșa and Cernat (2025) found that self-efficacy, together with personality traits and rational beliefs, significantly predicted academic performance among Romanian students, underscoring the cross-domain relevance of personal resource factors for children’s and adolescents’ adaptive functioning. Together, these factors reflect adaptive systems that have been preserved through the biological and sociocultural evolution of the human species. A similar perspective is presented by Ungar et al. (2019), who identified seven resources associated with positive developmental outcomes: access to material resources, supportive relationships, a positive personal identity, experiences of power and control, adherence to cultural traditions, experiences of social justice, and experiences of social cohesion.

The role of parents is further emphasized by Doty et al. (2017), who proposed the Cascading Resilience conceptual model, in which parenting interventions are regarded as a key mechanism for generating positive developmental cascades. Twum-Antwi et al. (2020) extended this framework by demonstrating that as caregivers become more resilient, children are more likely to benefit from the protective and promotive factors necessary for optimal development within both family and school environments. Relevant to the contextual variables examined in the present study, Howard and Johnson (2000), in a study involving children aged 9–12 years in South Australia, found that both children and teachers from socioeconomically disadvantaged communities identified family and community as two of the most important factors promoting resilience.

International evidence consistently demonstrates the relationship between socioeconomic status, parental education, and children’s resilience. Tok and Ünal (2020), in a sample of 384 five-year-old preschool children, reported a significant difference in resilience according to family income level (χ^2^ = 26.493, *p* < 0.05), with children from higher-income families demonstrating higher levels of resilience. They also found that children whose fathers had completed high school (M = 138.79) or university (M = 145.57) exhibited significantly higher resilience scores than children whose fathers had completed only primary education (M = 126.26; *F* = 7.517, *p* < 0.05). Several studies have also highlighted the importance of maternal education for children’s resilience. Longitudinal data from the Avon Longitudinal Study of Parents and Children (ALSPAC), based on 1117 mother–child dyads, showed that higher maternal educational attainment was associated with a stable trajectory of high resilience, whereas socioeconomic status did not independently predict this developmental pattern (Cahill et al., 2024). Similar findings have been reported in Indonesia, where parental education had a statistically significant effect (*p* = 0.046) (Caturulandari et al., 2025), and in Pakistan, where maternal education was associated with lower levels of stress and greater resilience among school-aged children (Mushtaq & Ali, 2024).

### 1.4. The Transmission of Parental Values and Children’s Socialization

Schwartz’s Theory of Basic Human Values (Schwartz, 1992), validated across numerous international samples through its structure of ten basic values and four higher-order value dimensions (Gross & Dewaele, 2018), has also been confirmed in children. Evidence supporting the applicability of the model has been provided by the Picture-Based Value Survey for Children, administered to a sample of 411 Spanish children (Álvaro et al., 2025), as well as by the Polish adaptation of the instrument (Cieciuch et al., 2013). Furthermore, the circular structure of Schwartz’s value model has been shown to be well established by middle childhood, although the hierarchy of values continues to evolve throughout childhood (Cieciuch et al., 2016). These findings support the relevance of Schwartz’s model for the age group examined in the present study.

The selection of parental values as a predictor of children’s resilience is grounded in the literature on intergenerational value transmission. Boehnke (2001), in a study involving 98 parent–student triads from East Germany, demonstrated that intergenerational value stability was stronger for self-transcendence than for self-enhancement, whereas conservation values were more strongly endorsed by parents. The transmission of values from parents to children is influenced not only by the type of values promoted but also by the quality of the parent–child relationship. Döring et al. (2017) found that parents’ prosocial educational goals contributed significantly to value similarity between parents and their children. Likewise, Vedder et al. (2009) reported that collectivist values are transmitted more frequently than individualist values, in line with Schwartz’s theoretical model, whereas Barni et al. (2011) demonstrated that the acceptance of parental values is facilitated by a close parent–child relationship.

An important consideration for interpreting the findings of the present study emerges from research on twins, which suggests that parent–child value similarity is determined primarily by shared genetic influences rather than solely by direct parental interaction (Kandler et al., 2016; Uzefovsky et al., 2016).

The relationship between parents’ value climate and children’s developmental outcomes has been directly documented by García et al. (2018), who demonstrated, in a large study of Spanish families, that indulgent and authoritative parenting styles were associated with stronger internalization of parental self-transcendence and conservation values, whereas authoritarian and neglectful parenting styles were associated with lower levels of value internalization. Palacios et al. (2022) complemented these findings by showing that positive parenting, combined with parental universalism values, protects children against emotional vulnerability. Similarly, Pastorelli et al. (2021), in a study conducted across 11 cultural groups, found that parents’ family values predicted adolescents’ prosocial behavior three years later through the internalization of those values by their children. Furthermore, Hadjar et al. (2012), analyzing data from 977 families in Germany and Israel, reported that greater value similarity between parents and adolescents was associated with higher life satisfaction. Finally, the relationship between values and resilience has been explicitly supported in the recent literature. Reparaz et al. (2021) emphasized that resilience education begins with the practice of parental values and is fostered through high-quality family interactions.

### 1.5. The Present Study

Against the background outlined above, the present study investigates the relationship between children’s resilience and two categories of factors: (a) socio-demographic factors, with a particular focus on parental educational attainment, socioeconomic status, family structure, and religious affiliation; and (b) parental values, operationalized according to Schwartz’s value model. Children’s resilience was assessed using the Child and Youth Resilience Measure (CYRM-28), a 28-item self-report questionnaire completed by the children. Parental values were assessed using the Short Schwartz’s Value Survey (SSVS), a 10-item questionnaire completed by one of the child’s parents, while demographic and socioeconomic data were collected through a parent-completed socio-demographic questionnaire. The proposed research design is consistent with Masten’s process-oriented conceptualization of resilience and with the multisystemic approach to resilience (Masten, 2018, 2019; Twum-Antwi et al., 2020).

The central hypothesis of the present study, namely that socio-normative parental values (security, conformity, tradition, universalism, and benevolence) are significantly associated with children’s resilience, whereas self-enhancement values (power and achievement) are not, is supported by previous research. García et al. (2018), for example, demonstrated that in families characterized by supportive parenting styles, self-transcendence and conservation values are more strongly internalized by children. Similarly, Pastorelli et al. (2021) found that parents’ familism values influence children’s prosocial behavior through a sequential mediation process, whereas Hadjar et al. (2012) reported that greater value similarity between parents and children is associated with higher levels of well-being. Likewise, Boehnke (2001) showed that intergenerational transmission is more stable for self-transcendence values than for self-enhancement values, thereby providing additional theoretical support for the proposed hypothesis.

#### Study Objectives and Hypotheses

The study pursued two main objectives: (1) to examine the associations between children’s self-reported resilience and socio-demographic and contextual factors, including parental educational attainment, socioeconomic status, family structure, religious affiliation, ethnicity, and age; and (2) to examine the specificity of the relationship between parental values and children’s resilience within Schwartz’s value framework. Based on the literature reviewed above, the following hypotheses were formulated. Because the evidence on the three contextual variables addressed in H3 is inconsistent and no comparable Romanian data are available (Section 1.2), that hypothesis is stated in non-directional terms:

**H1.**  *Higher parental educational attainment is positively associated with children’s resilience*.

**H2.**  *Socio-normative parental values (security, conformity, tradition, universalism, and benevolence) are positively associated with children’s resilience, whereas self-enhancement values and openness-to-change values are not significantly associated with children’s resilience*.

**H3.**  *Children’s resilience differs according to contextual variables, including religious affiliation, family structure, and ethnicity*.

**H4.**  *Children’s age is positively associated with their resilience, such that older children are expected to show higher levels of resilience*.

## 2. Materials and Methods

The present study employed a quantitative, cross-sectional, correlational design using child self-report and parent-report measures.

### 2.1. Participants

A non-probability convenience sampling strategy was employed, and the study was conducted in a rural setting. The final sample consisted of N = 245 children aged between 8 and 15 years (*M* = 10.58, *SD* = 1.95, median = 10). The age distribution was as follows: 8 years (18.0%), 9 years (17.6%), 10 years (20.0%), 11 years (8.6%), 12 years (13.9%), 13 years (14.3%), 14 years (6.8%), and 15 years (0.8%). Thus, the majority of participants were in middle childhood (9–13 years; 74.3%).

Regarding family characteristics, 75.1% of the participants came from intact families (children living with both biological parents), whereas 24.9% came from non-intact families (single-parent families, reconstituted families, in which the child lives with one parent and that parent’s new partner, and other arrangements). Most participants identified as Eastern Orthodox Christians (87.8%), 80.0% were of Romanian ethnicity, and 23.7% lived in households that included their grandparents. The distribution of parental educational attainment across the five educational levels was as follows: primary education (4 grades), 22.4%; lower secondary education (8 grades), 36.3%; upper secondary education (12 grades), 25.7%; university degree, 12.3%; and doctoral degree, 3.3%. For each participating child, one parent or primary caregiver completed the parent questionnaire, including the socio-demographic section (Table 1).

### 2.2. Measures

#### 2.2.1. Children’s Resilience—Child and Youth Resilience Measure (CYRM-28)

Children’s resilience was assessed using the Child and Youth Resilience Measure (CYRM-28), an internationally recognized instrument developed by Ungar and Liebenberg within the International Resilience Project (Ungar & Liebenberg, 2011) to assess three components of the resilience process: individual capacities, relationships with the primary caregiver, and contextual or sense-of-belonging factors. The instrument consists of 28 items rated on a five-point Likert scale. The total resilience score is calculated by summing all item scores (possible range: 28–140), with higher scores indicating higher levels of resilience. The questionnaire was completed directly by the children.

In the present sample, the CYRM-28 demonstrated excellent internal consistency for the total score (Cronbach’s α = 0.926, 95% CI [0.912, 0.938]) and good reliability for the three canonical subscales: Individual Capacities (11 items, α = 0.858, 95% CI [0.830, 0.883]), Relationship with Primary Caregiver (7 items, α = 0.783, 95% CI [0.739, 0.822]), and Contextual Factors/Sense of Belonging (10 items, α = 0.818, 95% CI [0.782, 0.850]).

At the time of data collection, no published validation of the CYRM-28 was available for the Romanian population. Therefore, the instrument was translated and culturally adapted into Romanian using the standard forward–backward translation procedure, in accordance with the approach recommended by the scale developers, who encourage the adaptation of the instrument to local contexts (Ungar & Liebenberg, 2011), as well as with the meta-analytic recommendation that the psychometric properties of the instrument should be re-evaluated in each national context (Renbarger et al., 2020). The internal consistency reported above supports the reliability of the Romanian version used in this study. Nevertheless, a comprehensive psychometric validation of the Romanian version remains an important objective for future research.

#### 2.2.2. Parental Values—Short Schwartz’s Value Survey (SSVS)

Parental values were assessed using the Romanian version of the Short Schwartz’s Value Survey (SSVS; Lindeman & Verkasalo, 2005), adapted and validated for the Romanian population by Voicu and Voicu (2007). The instrument measures the ten basic values proposed in Schwartz’s theory (Schwartz, 1992): *Power, Achievement, Hedonism, Stimulation, Self-Direction, Universalism, Benevolence, Tradition, Conformity*, and *Security*. Each was assessed by a single item accompanied by the corresponding value descriptors. Respondents rated the importance of each value as a guiding principle in their lives using a nine-point scale. The questionnaire was completed by one of the child’s parents.

The SSVS is a brief instrument designed to provide an efficient assessment of personal values while reducing administration time and respondents’ cognitive burden (Stern et al., 1998; Lindeman & Verkasalo, 2005; Sandy et al., 2017; Costa et al., 2023). Validation studies have confirmed the instrument’s quasi-circumplex structure, consistent with Schwartz’s theoretical model, its high congruence with the full Schwartz Value Survey (congruence coefficient ≈ 0.96) and the Portrait Values Questionnaire, as well as its satisfactory test–retest reliability (Lindeman & Verkasalo, 2005).

Because each basic value is assessed by a single item, internal consistency cannot be estimated for the individual value scales. Therefore, the analyses were conducted both at the level of the individual values and at the level of the higher-order value dimensions, computed as the mean scores of their constituent values on the original nine-point scale, without prior standardization: Self-Transcendence (Universalism, Benevolence), Self-Enhancement (Power, Achievement), Conservation (Security, Conformity, Tradition), and Openness to Change (Self-direction, Stimulation, Hedonism). In the present study, the term socio-normative values is used as a conceptual label for five basic values: Security, Conformity, Tradition, Universalism, and Benevolence. These values were analysed individually. In addition, two composite scores corresponding to the higher-order dimensions were computed separately: Conservation, as the mean of Security, Conformity and Tradition, and Self-Transcendence, as the mean of Universalism and Benevolence. No single composite score was computed or analysed for the socio-normative values as a whole. Because parental values and children’s resilience were reported by different respondents, the value scores were analyzed in their raw form, as controlling for response style was not considered necessary in a cross-source research design.

#### 2.2.3. Socio-Demographic Questionnaire

A structured parent-completed socio-demographic questionnaire was used to collect information on parental educational attainment (primary education, 4 grades; lower secondary education, 8 grades; upper secondary education, 12 grades; university degree, bachelor’s or master’s level; and doctoral degree), perceived family socioeconomic status, family structure, religious affiliation, number of children in the household, household composition, and co-residence with grandparents. For the analyses, family structure was dichotomized into intact families (children living with both biological parents; n = 184) and non-intact families (n = 61), comprising single-parent families and reconstituted families, in which the child lives with one biological parent and that parent’s new partner. Family structure was recorded in four categories: children living with both biological parents (n = 184), single-parent families (n = 29), reconstituted families, in which the child lives with one biological parent and that parent’s new partner (n = 23), and other arrangements (n = 9). For the analyses, these were dichotomized into intact families (n = 184) and non-intact families (n = 61). The four original categories were retained for the Kruskal–Wallis test and the post hoc comparisons reported in Section 3.2. Participants who selected “other arrangements” (n = 9) were included in the non-intact family category for the analyses, as they did not report living with both biological parents.

### 2.3. Procedure

The study protocol was implemented with the approval of the participating educational institutions. Children’s participation was based on informed parental consent and children’s assent, in accordance with the requirements of the General Data Protection Regulation (GDPR).

Data were collected between December 2025 and May 2026. The questionnaires administered to the children were completed face-to-face in the school setting under the supervision of the participating classroom teachers and a psychologist from the Coșa & Asociații Civil Professional Society of Psychology. Parent questionnaires were completed either at school or at home, depending on each family’s circumstances, and were subsequently returned to the research team through the classroom teachers or the psychologists.

### 2.4. Statistical Analysis

Prior to statistical analyses, the dataset underwent a data quality screening procedure, including the treatment of missing values and the computation of the total resilience score from the individual CYRM-28 items. The internal consistency of the CYRM-28 and of its subscales is reported in Section 2.2.1; Cronbach’s alpha was not computed for the Short Schwartz’s Value Survey, because each of its ten items is a distinct motivational value measured by a single item.

Because the distribution of the total resilience score deviated significantly from normality (Shapiro–Wilk W = 0.873, *p* < 0.001), the hypotheses were tested using nonparametric statistical methods, including Spearman’s rank-order correlation coefficient, the Kruskal–Wallis test, and the Mann–Whitney U test, with Holm’s correction applied to adjust for multiple comparisons. Differences across the five categories of parental educational attainment and across the four categories of family structure were examined further with the Kruskal–Wallis test, followed by Dunn’s pairwise post hoc comparisons with Holm’s correction for multiple testing. Effect sizes are reported as epsilon-squared for the omnibus tests and as the rank-biserial correlation for the pairwise comparisons. Predictors for the hierarchical multiple linear regression were selected on theoretical grounds, in accordance with the study hypotheses, rather than on the basis of statistical significance in the preliminary bivariate analyses. Block 1 comprised the socio-demographic variables specified in H1, H3 and H4 (children’s age, parental educational attainment, family structure, and religious affiliation), and Block 2 added the two socially focused higher-order value dimensions specified in H2 (Conservation and Self-Transcendence). Parental educational attainment was entered as an ordinal predictor, from 1 (primary education) to 5 (doctoral degree), in accordance with the ordering of the educational levels; this coding should be interpreted with caution, since the doctoral category was small (n = 8). The ordinal coding was retained after a supplementary model with dummy-coded education required three additional degrees of freedom for a marginal gain in explained variance. This coding decision was verified with a supplementary model in which parental educational attainment was entered as a set of dummy variables, with primary education as the reference category; the results of that model are reported in Section 3.3. Co-residence with grandparents was not included because it was not part of the prespecified model; its bivariate association is reported as exploratory.

**Figure 1:**
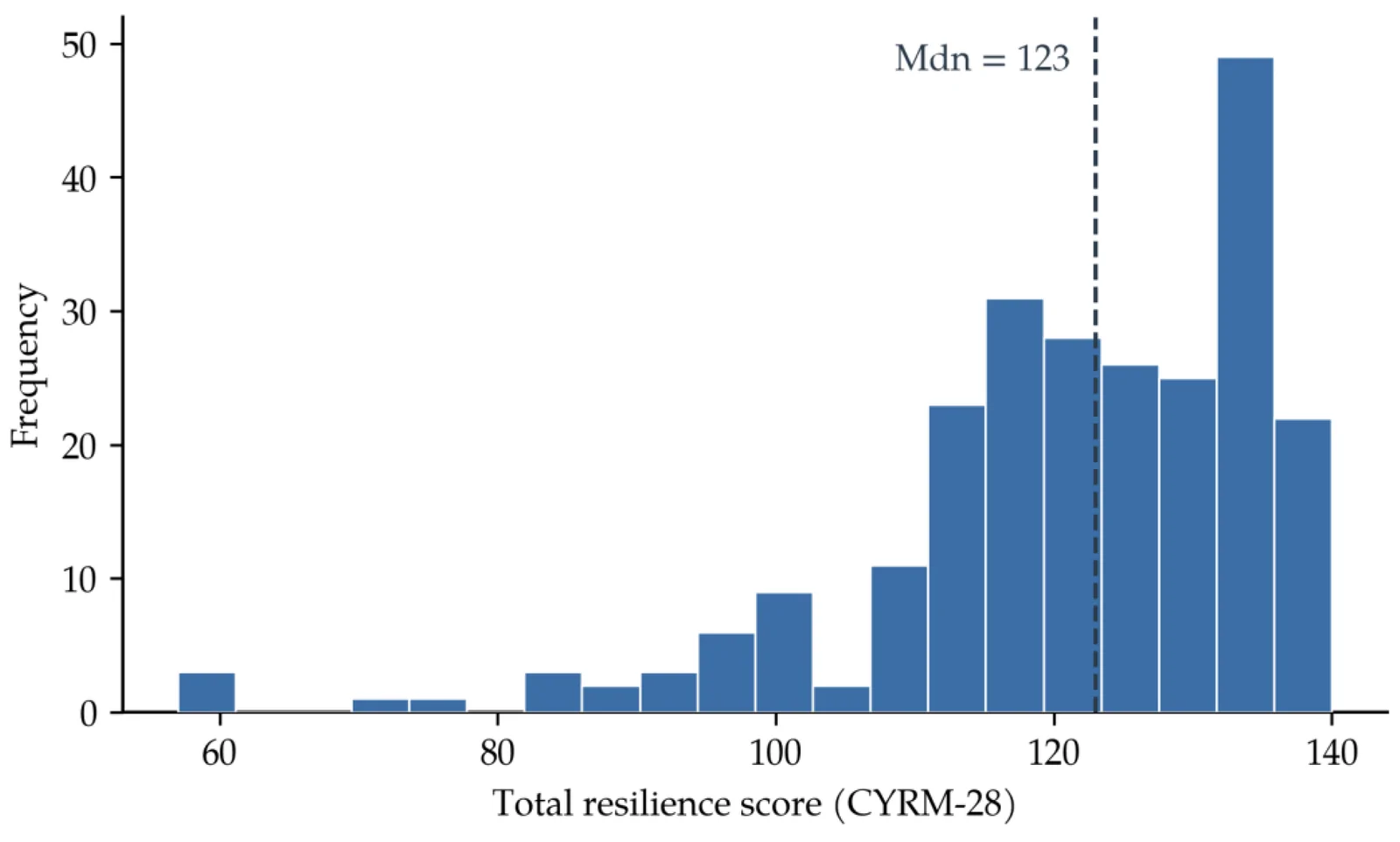
Distribution of the total resilience score (CYRM-28). The dashed line indicates the median (*Mdn* = 123).

As shown in Figure 1, the distribution of resilience scores was skewed toward the upper end of the scale.

These variables were entered into a hierarchical multiple linear regression model with children’s resilience as the dependent variable. Robust HC3 standard errors were used to account for the non-normality and heteroscedasticity of the residuals. Statistical analyses were performed using IBM SPSS Statistics (Version 23), whereas the hierarchical regression model with HC3 robust standard errors was estimated in Python (Version 3.12) using the statsmodels library.

## 3. Results

### 3.1. Descriptive Statistics

The mean resilience score was 120.88 (*SD* = 14.89, *Mdn* = 123, range = 57–140), corresponding to a mean item score of 4.32 out of a maximum possible score of 5. These findings indicate a high level of self-reported resilience among the participating children.

Table 2 presents the mean scores for the individual values included in Schwartz’s value model, together with their correlations with children’s resilience.

### 3.2. Hypothesis Testing

**H1.**  *Parental educational attainment and children’s resilience*.

**Figure 2:**
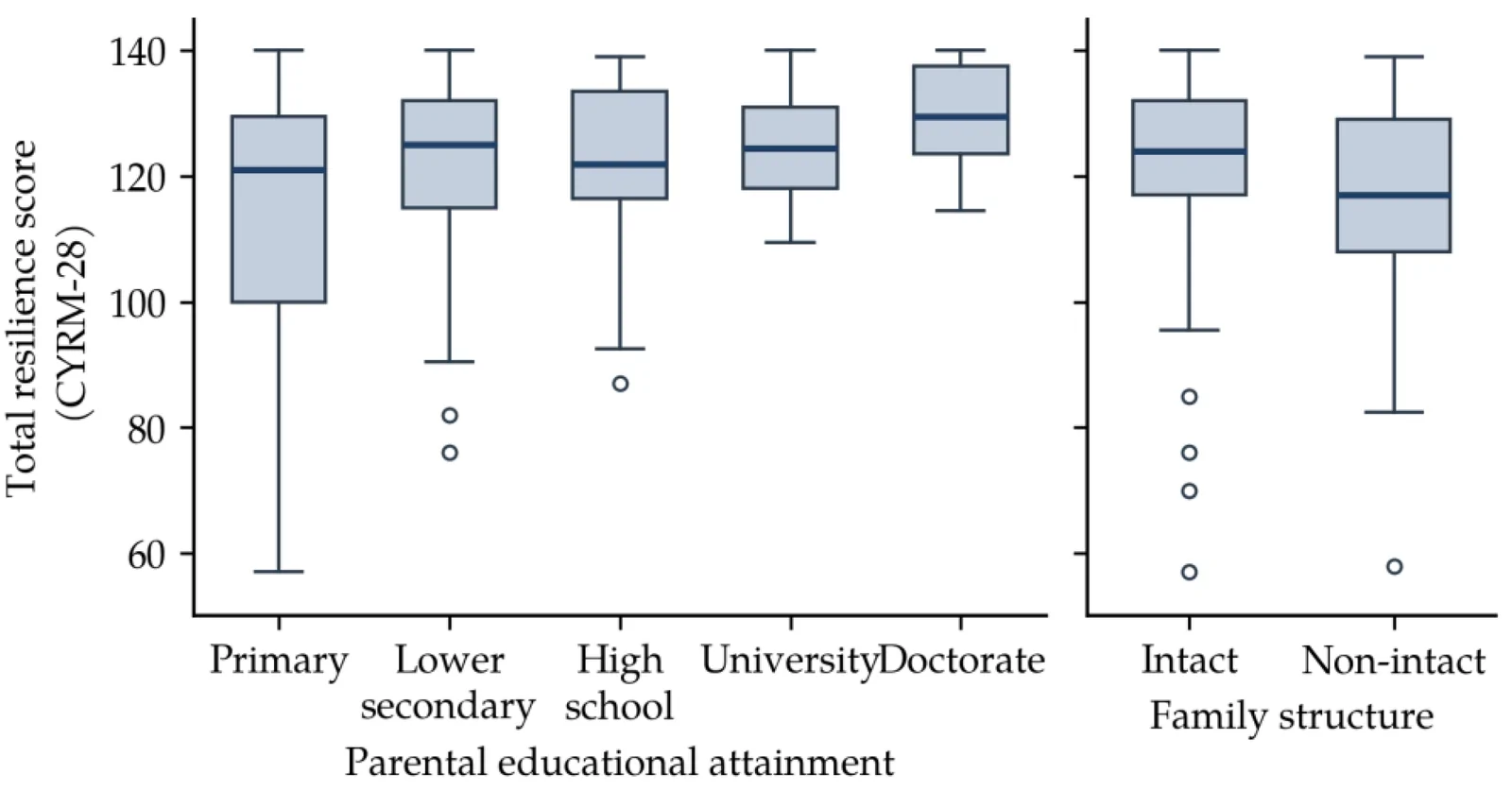
Distribution of children’s resilience scores according to parental educational attainment (**left panel**) and family structure (**right panel**), presented as boxplots.

The results indicated that higher parental educational attainment was positively associated with higher levels of children’s resilience (Spearman’s ρ = 0.188, *p* = 0.003). In addition, the Kruskal–Wallis test revealed significant differences in resilience across the five educational categories (*H*(4) = 11.52, *p* = 0.021, ε^2^ = 0.047). The median resilience score increased progressively from 121 among children whose parents had completed primary education to 129.5 among those whose parents held a doctoral degree (Figure 2 left panel). Therefore, H1 was supported, although the observed effect size was small. Dunn’s post hoc comparisons with Holm’s correction revealed no significant differences between any pair of categories; the largest contrast, between primary education and the doctoral degree, did not survive correction (z = −2.63, *p* = 0.009, p_holm_ = 0.086). The association across educational levels therefore takes the form of a monotonic trend rather than a difference between specific pairs of categories. The absence of significant pairwise contrasts does not by itself establish a graded pattern across the educational levels. The evidence for an association rests on the ordinal Spearman correlation, and the present data do not allow its shape to be characterized more precisely. Given the small size of the doctoral-degree subgroup (n = 8; 3.3% of the sample), findings involving this category should be interpreted with caution. As a sensitivity check, the post hoc comparisons were repeated with the doctoral and university categories combined into a single higher-education group (n = 38). In that analysis, the contrast between primary education and higher education reached significance (z = −3.00, p_holm_ = 0.016, r = −0.35), indicating that the absence of significant pairwise differences in the five-category analysis reflects in part the limited size of the two highest categories rather than the absence of a difference between educational levels.

**H2.**  *Schwartz’s individual values and children’s resilience*.

Six of the ten basic values were positively and significantly correlated with children’s resilience, even after applying Holm’s correction for multiple comparisons: Security (ρ = 0.316), Universalism (ρ = 0.250), Benevolence (ρ = 0.225), Conformity (ρ = 0.202), Self-Direction (ρ = 0.198), and Tradition (ρ = 0.189). In contrast, the self-enhancement and openness-to-change values (Power, Achievement, Hedonism, and Stimulation) were not significantly associated with resilience.

At the level of the higher-order value dimensions, Conservation (ρ = 0.306, *p* < 0.001) and Self-Transcendence (ρ = 0.275, *p* < 0.001) were positively associated with children’s resilience, whereas Self-Enhancement (ρ = 0.067) and Openness to Change (ρ = 0.055) showed no significant associations.

**Figure 3:**
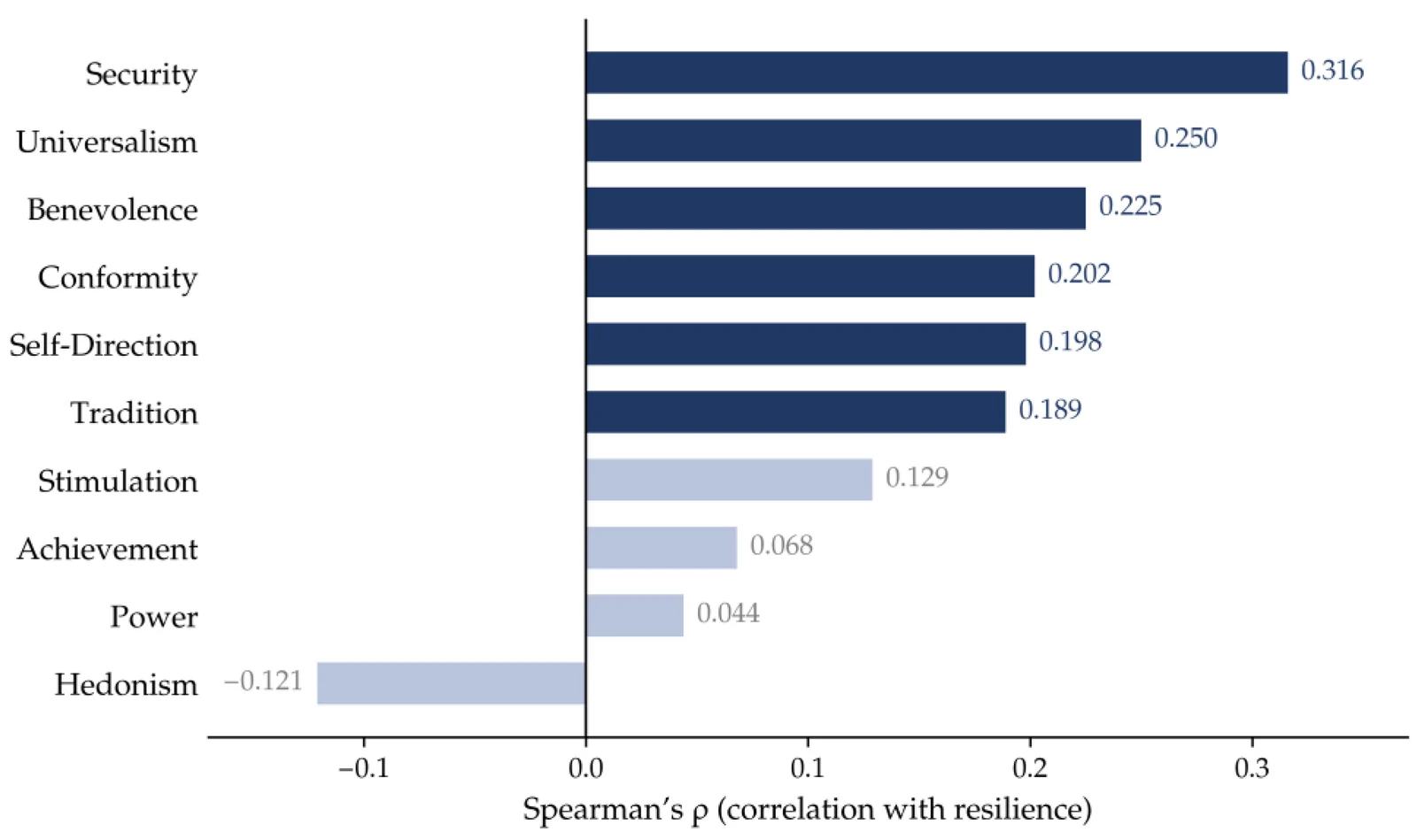
Spearman correlations between Schwartz’s individual values and children’s resilience. Dark bars indicate statistically significant associations after Holm’s correction (*p* < 0.05); light bars indicate non-significant associations.

Thus, H2 received partial support, as the observed relationships were specific to the socially focused values of Schwartz’s model, Conservation and Self-Transcendence (Table 2; Figure 3).

**H3.**  *Group differences according to religious affiliation, ethnicity, and family structure*.

Significant differences in children’s resilience were observed according to religious affiliation (*Mdn* = 124 for Eastern Orthodox children vs. *Mdn* = 117.5 for children belonging to other religious denominations; Mann–Whitney *U* = 4227, *p* = 0.006) and family structure (*Mdn* = 124 for children from intact families vs. *Mdn* = 117 for children from non-intact families; Mann–Whitney *U* = 7074, *p* = 0.002; Figure 3, right panel). The Kruskal–Wallis test conducted across the four family structure categories also yielded significant differences (*H*(3) = 10.84, *p* = 0.013, ε^2^ = 0.044). Dunn’s post hoc comparisons with Holm’s correction located this difference in a single contrast, between children living with both parents and children from reconstituted families (n = 23; Mdn = 116 vs. 124; z = 2.91, p_holm_ = 0.022, r = 0.38); children from single-parent families did not differ significantly from children living with both parents (p_holm_ = 0.66). Given the small size of that subgroup, this refinement of the binary comparison should be regarded as provisional.

No significant differences in resilience were found according to ethnicity (*p* = 0.717).

Accordingly, H3 received partial support, being confirmed for religious affiliation and family structure but not for ethnicity (Table 3).

**H4.**  *Children’s age and resilience*.

Contrary to expectations, the hypothesis of a positive association between age and resilience, in the sense that older children would show higher levels of resilience, was not supported. Age was negatively correlated with children’s resilience (Spearman’s ρ = −0.228, *p* < 0.001), indicating that younger children reported slightly higher resilience scores than older children. Therefore, the findings do not support the assumption that resilience increases progressively with age during childhood and early adolescence.

#### Additional Socio-Demographic Associations

Perceived socioeconomic status (ρ = 0.005), household size (ρ = −0.104), and the number of children in the family (ρ = −0.043) were not significantly associated with children’s resilience. However, children who did not live with their grandparents reported higher median resilience scores than those living with their grandparents (*Mdn* = 124 vs. 118; Mann–Whitney *U* = 6633, *p* = 0.010).

### 3.3. Hierarchical Multiple Regression Analysis

The regression residuals exhibited both non-normality and heteroscedasticity (Breusch–Pagan test, *p* < 0.001), and multicollinearity was negligible, with all variance inflation factors below 1.9.

The regression analysis showed that the socio-demographic variables explained 15.0% of the variance in children’s resilience (*R*^2^ = 0.150, *p* < 0.001). The inclusion of the higher-order value dimensions significantly improved the model, accounting for an additional 7.6% of the explained variance (Δ*R*^2^ = 0.076, *F*(2, 238) = 11.76, *p* < 0.001). The final model explained 22.6% of the variance in children’s resilience (adjusted *R*^2^ = 0.207).

After all predictors had been entered into the model, parental educational attainment (β = 0.256), Conservation values (β = 0.191), Eastern Orthodox religious affiliation (β = 0.167), and living in an intact family (β = 0.136) remained significant predictors of children’s resilience. In contrast, children’s age and Self-Transcendence values were no longer statistically significant predictors (Table 4).

As a robustness check, the final model was re-estimated with parental educational attainment represented by four dummy variables, with primary education as the reference category. Compared with primary education, the coefficients were positive and statistically significant for lower secondary education (b = 8.52, robust SE = 2.72, *p* = 0.002), upper secondary education (b = 9.21, robust SE = 2.91, *p* = 0.002), university studies (b = 12.72, robust SE = 3.20, *p* < 0.001) and doctoral studies (b = 14.03, robust SE = 3.62, *p* < 0.001). The categorical model explained 24.4% of the variance (R^2^ = 0.244; adjusted R^2^ = 0.215), compared with 22.6% in the ordinal model (R^2^ = 0.226; adjusted R^2^ = 0.207), an increase of 1.8 percentage points in explained variance for three additional degrees of freedom. The direction and the statistical significance of the remaining predictors were unchanged. The substantive conclusions therefore did not depend appreciably on the way parental educational attainment was coded.

## 4. Discussion

The present study examined the relationship between children’s resilience and three categories of factors, socio-demographic characteristics, contextual factors, and parental values based on Schwartz’s Theory of Basic Human Values, in a Romanian sample. This represents a particularly relevant context, as empirical research on children’s resilience in Romania remains limited (Ungar et al., 2008). Overall, the findings reveal a coherent pattern: higher levels of children’s resilience were associated with greater parental educational attainment, an intact family structure, Eastern Orthodox religious affiliation, and, most notably, the conceptual grouping of socio-normative values (Conservation and Self-Transcendence). These findings are discussed below in light of the theoretical frameworks that informed the present study.

### 4.1. Parental Educational Attainment and Children’s Resilience

The positive association between parental educational attainment and children’s resilience, although modest in magnitude, is consistent with previous research identifying parental resources, such as support, monitoring, and effective communication, as key protective factors for children’s adaptive development (Zolkoski & Bullock, 2012). Higher parental educational attainment may serve as an indirect indicator of a cognitively stimulating home environment, more structured parenting practices, and greater access to educational and social resources. These mechanisms are consistent with the Cascading Resilience model proposed by Doty et al. (2017), according to which parenting practices constitute a central mechanism through which positive developmental cascades can be initiated.

The relatively small effect size is also consistent with Masten’s (2001) concept of ordinary magic, which emphasizes that resilience does not arise from rare or exceptional protective factors but from the cumulative influence of ordinary adaptive resources available in children’s everyday environments. From this perspective, no single protective factor is expected to exert a dominant influence. Rather, resilience emerges through the combined effects of multiple adaptive processes operating across interconnected biological, psychological, familial, and social systems.

### 4.2. The Parental Value Profile Associated with Children’s Resilience

All five basic values included in the conceptual grouping of socio-normative values, Security, Conformity, Tradition, Universalism, and Benevolence, were significantly associated with children’s resilience. Self-Direction, an Openness to Change value with a predominantly personal focus, also showed a significant association (ρ = 0.198), whereas Power, Achievement, Hedonism, and Stimulation were not significantly associated with resilience. The higher-order dimension Openness to Change as a whole was not significantly associated with resilience (ρ = 0.055).

Viewed through the lens of socialization theory, this pattern suggests that children’s resilience is associated not with parental values in general, but specifically with parents’ prioritization of socio-normative values, most likely through the family climate shaped by these value orientations. The finding that Security showed the strongest association with children’s resilience (ρ = 0.316) is consistent with Masten’s (2018) systemic definition of resilience as the capacity of a dynamic system to adapt successfully to challenges that threaten its functioning. A family environment that emphasizes security, stability, and predictability provides precisely the conditions that enable children to maintain adaptive functioning under stress (Leckman, 2007). This interpretation is further supported by the Relationship with Primary Caregiver subscale of the CYRM-28, which directly captures this relational dimension of resilience.

Similarly, the positive association between parental Self-Transcendence values (Universalism and Benevolence) and children’s resilience highlights the fundamentally relational nature of resilience. Through modeling and socialization, parents who prioritize prosocial values are more likely to foster the “ordinary protective systems” identified by Masten (2001), namely supportive relationships and a sense of community belonging, which constitute the foundation of resilient adaptation and are operationalized in the relational and contextual subscales of the CYRM-28. This interpretation is fully consistent with Ungar’s ecological and cultural perspective on resilience (Ungar, 2008; Ungar & Liebenberg, 2011), according to which resilience depends on the opportunities and resources available and accessible to children within their everyday environments. From this perspective, the protective factors underlying resilience are shaped by the values and cultural norms of the communities in which children develop. Accordingly, socio-normative parental values may be understood as indicators of the family’s integration into broader networks of meaning, belonging, and social responsibility. This interpretation is also consistent with Panter-Brick’s observation (Southwick et al., 2014) that resilience is guided by culturally specific goals and encompasses moral and social dimensions that extend beyond individual well-being.

Two additional considerations deserve attention. First, in the multivariate analysis, after controlling for socio-demographic characteristics, only Conservation remained independently associated with children’s resilience (β = 0.191, *p* = 0.028), whereas Self-Transcendence no longer showed an independent association (β = 0.118, *p* = 0.149). Thus, the robust association observed for the conceptual grouping of socio-normative values appears to be driven primarily by Conservation, particularly Security, Conformity, and Tradition. Second, the correlational nature of the data requires caution in interpreting these findings. A reciprocal relationship is plausible, whereby parental value orientations and children’s resilience mutually reinforce one another throughout development. Nevertheless, one important methodological strength should be emphasized: because parental values were reported by parents, whereas children’s resilience was self-reported, the observed associations cannot be attributed to common method variance, thereby increasing confidence in the robustness of the findings.

### 4.3. Family and Religious Context

The finding that children from intact families reported higher levels of resilience supports the proposition that the effects of individual adversity are amplified by cumulative environmental challenges (Sameroff & Rosenblum, 2006) and that resilience is shaped by parenting processes and social support (Smith & Carlson, 1997). An intact family structure may provide a more stable, predictable, and supportive caregiving environment, a dimension that is directly reflected in the Relationship with Primary Caregiver subscale of the CYRM-28. These caregiving processes were not assessed directly, so the observed difference cannot establish which family experiences account for it.

One possible explanation, not directly tested in the present study, is that religious affiliation indexes children’s access to a shared system of meaning and to a supportive community; these mechanisms were not measured, however, and alternative explanations (for example, socio-demographic differences between denominational groups) cannot be excluded. This interpretation is consistent with the protective role of value systems in sustaining meaning in life (Rak & Patterson, 1996; Werner, 1989) and with Clauss-Ehlers’s (2008, as cited in Johns et al., 2014) argument for culturally sensitive approaches to resilience assessment. Notably, this finding also aligns with the parental value profile identified in the present study, as religious affiliation and the values of Tradition and Conformity belong to the same conceptual grouping of socio-normative values.

The absence of significant differences according to ethnicity may reflect both the relatively small and heterogeneous minority subsamples and the ongoing tension between within-group homogeneity and between-group heterogeneity highlighted by Ungar (2015). This null result is exploratory and should be interpreted with caution, given the size and the heterogeneity of the minority subsample (n = 49); it does not establish the absence of ethnic or cultural variation in resilience.

### 4.4. Age and Children’s Resilience: An Unsupported Hypothesis

Contrary to expectations, older age was not associated with higher levels of children’s resilience. Instead, the results revealed a modest negative association, with younger children reporting slightly higher resilience scores than their older peers. However, this finding should not be interpreted as evidence that resilience declines with age. Given the cross-sectional design of the study, no conclusions can be drawn regarding developmental changes in resilience or the progressive consolidation of coping resources throughout middle childhood. Such developmental trajectories can only be adequately examined through longitudinal research (Werner, 1989; Rak & Patterson, 1996).

Moreover, the observed differences may reflect age-related differences in self-perception, as younger children may evaluate their own functioning in a more positive and less critical manner than older children; this explanation was not tested in the present study. The ceiling effect observed in the distribution of resilience scores may also have played a part in this pattern. Taken together, these findings support the view that resilience is a dynamic, context-dependent process (Soare, 2025), rather than a characteristic that increases linearly with age.

### 4.5. Theoretical and Practical Implications

From a theoretical perspective, the present study contributes to the literature by integrating Schwartz’s Theory of Basic Human Values with resilience research. The findings indicate that children’s resilience is associated not with parental values in general, but specifically with Conservation and Self-Transcendence values. This result reinforces the ecological and cultural perspectives on resilience (Ungar, 2008; Southwick et al., 2014) within the Romanian context, which has received limited attention in previous research.

From a practical perspective, the findings are consistent with McDonald et al. (2019), who argued that educating parents about the factors that promote children’s resilience is essential for the effectiveness of intervention programs. Accordingly, resilience-promoting initiatives may benefit from fostering children’s sense of security, belonging, and prosocial orientation while also providing additional support to parents with lower educational attainment, given the observed association between parental education and children’s resilience and its implications for educational equity.

### 4.6. Limitations and Future Research

The findings of the present study should be interpreted in light of several limitations. First, the cross-sectional and correlational design does not allow causal inferences to be drawn, nor does it permit an examination of how resilience and parental values develop over time. Longitudinal studies are therefore needed to confirm and extend the present findings.

Second, although the study relied on reports from two different informants (children completed the resilience measure, whereas parents completed the values questionnaire and the socio-demographic questionnaire), each construct was assessed using a single reporting source. Consequently, the findings may still have been influenced by social desirability and other response biases, which may partly explain the ceiling effect observed for the resilience scores.

Third, the sample was relatively homogeneous, consisting predominantly of Romanian children of Eastern Orthodox religious affiliation living in rural communities. In addition, the minority subgroups were relatively small, limiting the generalizability of the findings and reducing the statistical power of between-group comparisons. The same applies to the doctoral-degree category, which comprised only eight parents (3.3% of the sample); comparisons involving this group should therefore be treated as provisional. The ethnic-minority subsample (n = 49) was likewise small and internally heterogeneous, so the null result for ethnicity should be regarded as exploratory.

Fourth, the instrument used to assess children’s resilience has not yet undergone a comprehensive psychometric validation in the Romanian population. Although it demonstrated excellent internal consistency in the present sample, additional research is needed to establish its psychometric properties within the Romanian cultural context.

Fifth, parental values were reported by one parent or primary caregiver per child; in two-parent families, the values of the other parent were not assessed, so the broader parental value environment of the family was not fully captured. The respondent’s specific role (mother, father, or other caregiver) was not retained in the dataset, so its distribution and its possible influence on the reported values could not be examined.

Sixth, each SSVS value was measured by a single item, which limits the precision and the depth of value measurement and prevents the estimation of internal consistency for the individual values.

Finally, the cultural dimension was operationalized exclusively through Schwartz’s individual value framework, without incorporating broader societal or community-level cultural indicators. Future research could extend these findings by adopting longitudinal designs, incorporating assessments from multiple informants (e.g., parents and teachers), including both parents where applicable, employing comprehensive multi-item measures of values grounded in Schwartz’s theoretical framework (for example, the PVQ-RR), and including samples that are more diverse with respect to cultural background and residential setting. Building on such designs, conditional process models (e.g., moderated mediation) could further identify the mechanisms through which parental values are transmitted into children’s resilience, as well as the contextual conditions under which these pathways operate, an analytical approach recently applied to Romanian educational samples (Roman et al., 2026).

## 5. Conclusions

The present study contributes to the growing body of research on children’s resilience by highlighting the role of family context and parental values in a Romanian sample. Children’s resilience was positively associated with parental educational attainment, living in an intact family, Eastern Orthodox religious affiliation, and, most notably, with parental values emphasizing Conservation, Universalism, and Benevolence. In contrast, older age was not associated with higher levels of resilience.

The integrated model explained approximately one-fifth of the variance in children’s resilience, suggesting that parental values are associated with children’s resilience beyond socio-demographic factors. Although these findings require confirmation through longitudinal research and more diverse samples, they underscore the importance of family resources and parental value orientations for understanding and promoting children’s resilience.

## Figures and Tables

**Table 1:** Socio-demographic characteristics of the sample (N = 245).

Characteristic	n	%
**Parental educational attainment**		
Primary education (4 grades)	55	22.4
Lower secondary education (8 grades)	89	36.3
Upper secondary education (12 grades)	63	25.7
University degree (bachelor’s or master’s)	30	12.2
Doctoral degree	8	3.3
**Family structure**		
Intact families	184	75.1
Non-intact families	61	24.9
**Religious affiliation**		
Eastern Orthodox	215	87.8
Other denominations	30	12.2
**Ethnicity**		
Romanian	196	80.0
Ethnic minority	49	20.0
**Co-residence with grandparents**		
Yes	58	23.7
No	187	76.3
**Parent questionnaire respondent**		
One parent or primary caregiver per child	245	100.0

Note. One parent or primary caregiver completed the questionnaire for each child. The respondent role (mother, father, or other caregiver) was not retained.

**Table 2:** Descriptive Statistics for Schwartz’s Values and Spearman Correlations with Children’s Resilience.

Value	*M*	*SD*	ρ	Holm-Adjusted *p*
Power	5.46	1.95	0.044	0.583
Achievement	5.80	1.79	0.068	0.583
Hedonism	4.64	2.23	−0.121	0.177
Stimulation	5.35	1.97	0.129	0.176
Self-Direction	6.06	1.59	0.198 *	0.011
Universalism	6.22	1.61	0.250 **	0.001
Benevolence	6.64	1.41	0.225 **	0.003
Tradition	6.64	1.44	0.189 *	0.015
Conformity	6.83	1.44	0.202 *	0.010
Security	6.92	1.40	0.316 **	<0.001

Note. ρ = Spearman’s rank-order correlation coefficient with the total resilience score. Holm-adjusted *p*-values were calculated using Holm’s sequential correction for multiple comparisons. * *p* < 0.05; ** *p* < 0.01. The order of the values follows Schwartz’s canonical circumplex structure.

**Table 3:** Differences in Children’s Resilience According to Categorical Variables (Mann–Whitney U Test).

Variable (Grouping)	*Mdn* Group A	*Mdn* Group B	*U*	*p*
Religious affiliation (Eastern Orthodox vs. other)	124	117.5	4227	0.006
Family structure (intact vs. non-intact)	124	117	7074	0.002
Ethnicity (Romanian vs. minority)	123	125	4963	0.717

Note. To ensure the validity of the statistical analyses, categories with a small number of participants were combined into broader groups (ethnic minority, *n* = 49; non-Orthodox religious affiliation, *n* = 30; non-intact families, *n* = 61).

**Table 4:** Hierarchical Multiple Linear Regression Predicting Children’s Resilience (HC3 Robust Standard Errors).

Predictor	b	Robust SE	β	*p*	VIF
Children’s age	−0.01	0.49	−0.001	0.985	1.09
Parental educational attainment	3.59	0.86	0.256	<0.001	1.06
Intact family	4.66	2.17	0.136	0.031	1.16
Eastern Orthodox religious affiliation	7.59	3.13	0.167	0.015	1.14
Self-Transcendence	1.37	0.95	0.118	0.149	1.85
Conservation	2.43	1.11	0.191	0.028	1.90

Note. b = unstandardized regression coefficient; β = standardized regression coefficient; Robust SE = HC3 robust standard error; VIF = variance inflation factor. Final model: *R*^2^ = 0.226; adjusted *R*^2^ = 0.207; Δ*R*^2^ (Block 2) = 0.076; *p* < 0.001.

## Data Availability

The data supporting the findings of this study are available from the corresponding author upon reasonable request. The data are not publicly available because of privacy and confidentiality considerations.

## References

[B1-behavsci-16-01623] Álvaro J. L., Pascual Sánchez E., da Costa Silva K., Torres A. R., Garrido A., Rodríguez M. (2025). Validation of Schwartz’s human values scale in Spanish children. Psicología Educativa.

[B2-behavsci-16-01623] Barni D., Ranieri S., Scabini E., Rosnati R. (2011). Value transmission in the family: Do adolescents accept the values their parents want to transmit?. Journal of Moral Education.

[B3-behavsci-16-01623] Belsky J., Pluess M. (2009). Beyond diathesis stress: Differential susceptibility to environmental influences. Psychological Bulletin.

[B4-behavsci-16-01623] Blessin M., Lehmann S., Kunzler A. M., van Dick R., Lieb K. (2022). Resilience interventions conducted in Western and Eastern countries—A systematic review. International Journal of Environmental Research and Public Health.

[B5-behavsci-16-01623] Boehnke K. (2001). Parent-offspring value transmission in a societal context: Suggestions for a utopian research design—With empirical underpinnings. Journal of Cross-Cultural Psychology.

[B6-behavsci-16-01623] Cahill S., Hager R., Shryane N. (2024). Patterns of resilient functioning in early life: Identifying distinct groups and associated factors. Development and Psychopathology.

[B7-behavsci-16-01623] Caturulandari C., Kur’ani N., Ramadhan R. (2025). Studi perbandingan dampak pendidikan orang tua terhadap resiliensi siswa Madrasah Aliyah [A comparative study of the impact of parental education on the resilience of Madrasah Aliyah students]. PAEDAGOGY: Jurnal Ilmu Pendidikan dan Psikologi.

[B8-behavsci-16-01623] Cicchetti D., Garmezy N. (1993). Prospects and promises in the study of resilience. Development and Psychopathology.

[B9-behavsci-16-01623] Cicchetti D., Rogosch F. A. (2012). Gene × environment interaction and resilience: Effects of child maltreatment and serotonin, corticotropin releasing hormone, dopamine, and oxytocin genes. Development and Psychopathology.

[B10-behavsci-16-01623] Cieciuch J., Davidov E., Algesheimer R. (2016). The stability and change of value structure and priorities in childhood: A longitudinal study. Social Development.

[B11-behavsci-16-01623] Cieciuch J., Harasimczuk J., Döring A. K. (2013). Structural validity of the Polish adaptation of the Picture-Based Value Survey for Children (PBVS-C). Journal of Psychoeducational Assessment.

[B12-behavsci-16-01623] Ciren Z., Tsui H. K. H., Chan S. K. W. (2026). Effects of resilience interventions for adolescents and young adults without psychiatric diagnoses: A systematic review and network meta-analysis of randomized controlled trials. Adolescent Research Review.

[B13-behavsci-16-01623] Collins P. R., Sneddon J., Lee J. A. (2024). Personal values, subjective wellbeing, and the effects of perceived social support in childhood: A pre-registered study. European Journal of Psychology of Education.

[B14-behavsci-16-01623] Costa P. J. C., Moreira P. A. S., Faria S., Correia Lopes J. (2023). The Twenty Item Values Inventory (TwIVI) in Portuguese adults: Factorial structure, internal consistency, and criterion-related validity. Journal of Personality Assessment.

[B15-behavsci-16-01623] Coșa L. E., Cernat V. (2025). Exploring the predictors of academic performance: The role of personality, rational beliefs, and self-efficacy. Frontiers in Psychology.

[B16-behavsci-16-01623] Doty J. L., Davis L., Arditti J. A. (2017). Cascading resilience: Leverage points in promoting parent and child well-being. Journal of Family Theory & Review.

[B17-behavsci-16-01623] Döring A. K., Makarova E., Herzog W., Bardi A. (2017). Parent–child value similarity in families with young children: The predictive power of prosocial educational goals. British Journal of Psychology.

[B18-behavsci-16-01623] Fismen A.-S., Smith O. R. F., Helleve A., Haug E., Chatelan A., Kelly C., Dzielska A., Nardone P., Melkumova M., Ercan O., Kopcakova J., Lazzeri G., Klepp K.-I., Samdal O. (2022). Cross-national variation in the association between family structure and overweight and obesity: Findings from the Health Behaviour in School-aged Children (HBSC) study. SSM–Population Health.

[B19-behavsci-16-01623] García O. F., Serra E., Zacarés J. J., García F. (2018). Parenting styles and short- and long-term socialization outcomes: A study among Spanish adolescents and older adults. Psychosocial Intervention.

[B20-behavsci-16-01623] Gross B., Dewaele J.-M. (2018). The relation between multilingualism and basic human values among primary school children in South Tyrol. International Journal of Multilingualism.

[B21-behavsci-16-01623] Hadjar A., Boehnke K., Knafo A., Daniel E., Musiol A.-L., Schiefer D., Möllering A. (2012). Parent-child value similarity and subjective well-being in the context of migration: An exploration. Family Science.

[B22-behavsci-16-01623] Howard S., Johnson B. (2000). What makes the difference? Children and teachers talk about resilient outcomes for children ‘at risk’. Educational Studies.

[B23-behavsci-16-01623] Johns A., Grossman M., McDonald K. (2014). ‘More than a game’: The impact of sport-based youth mentoring schemes on developing resilience toward violent extremism. Social Inclusion.

[B24-behavsci-16-01623] Kandler C., Gottschling J., Spinath F. M. (2016). Genetic and environmental parent–child transmission of value orientations: An extended twin family study. Child Development.

[B25-behavsci-16-01623] Leckman J. F. (2007). Genes and behavior: Nature–nurture interplay explained. Journal of Child Psychology and Psychiatry.

[B26-behavsci-16-01623] Lindeman M., Verkasalo M. (2005). Measuring values with the Short Schwartz’s Value Survey. Journal of Personality Assessment.

[B27-behavsci-16-01623] Marks A. K., Woolverton G. A., García Coll C. (2020). Risk and resilience in minority youth populations. Annual Review of Clinical Psychology.

[B28-behavsci-16-01623] Masten A. S. (2001). Ordinary magic: Resilience processes in development. American Psychologist.

[B29-behavsci-16-01623] Masten A. S. (2018). Resilience theory and research on children and families: Past, present, and promise. Journal of Family Theory & Review.

[B30-behavsci-16-01623] Masten A. S. (2019). Resilience from a developmental systems perspective. World Psychiatry.

[B31-behavsci-16-01623] Masten A. S., Best K. M., Garmezy N. (1990). Resilience and development: Contributions from the study of children who overcome adversity. Development and Psychopathology.

[B32-behavsci-16-01623] Masten A. S., Obradović J. (2008). Disaster preparation and recovery: Lessons from research on resilience in human development. Ecology and Society.

[B33-behavsci-16-01623] McDonald M., McCormack D., Avdagic E., Hayes L., Phan T., Dakin P. (2019). Understanding resilience: Similarities and differences in the perceptions of children, parents and practitioners. Children and Youth Services Review.

[B34-behavsci-16-01623] Mushtaq A., Ali S. M. (2024). Investigating the moderating contribution of mothers’ educational level with resilience and stress among school children. Academy of Education and Social Sciences Review.

[B35-behavsci-16-01623] Nguyen K., Stanley N., Rank A., Stanley L., Wang Y. (2016). The relationship among storytelling, values, and resilience of college students from Eastern and Western cultural backgrounds. Journal of Poetry Therapy.

[B36-behavsci-16-01623] Palacios I., Garcia O. F., Alcaide M., Garcia F. (2022). Positive parenting style and positive health beyond the authoritative: Self, universalism values, and protection against emotional vulnerability from Spanish adolescents and adult children. Frontiers in Psychology.

[B37-behavsci-16-01623] Pastorelli C., Zuffianò A., Lansford J. E., Thartori E., Bornstein M. H., Chang L., Deater-Deckard K., Di Giunta L., Dodge K. A., Gurdal S., Liu Q., Long Q., Oburu P., Skinner A. T., Sorbring E., Steinberg L., Tapanya S., Uribe Tirado L. M., Yotanyamaneewong S., Bacchini D. (2021). Positive youth development: Parental warmth, values, and prosocial behavior in 11 cultural groups. Journal of Youth Development.

[B38-behavsci-16-01623] Pooley J. A., Cohen L. (2010). Resilience: A definition in context. The Australian Community Psychologist.

[B39-behavsci-16-01623] Rak C. F., Patterson L. E. (1996). Promoting resilience in at-risk children. Journal of Counseling & Development.

[B40-behavsci-16-01623] Renbarger R. L., Padgett R. N., Cowden R. G., Govender K., Yilmaz M. Z., Scott L. M., Makhnach A. V., Novotny J. S., Nugent G., Rosenbaum L., Křeménková L. (2020). Culturally relevant resilience: A psychometric meta-analysis of the Child and Youth Resilience Measure (CYRM). Journal of Research on Adolescence.

[B41-behavsci-16-01623] Reparaz C., Rivas S., Osorio A., Garcia-Zavala G. (2021). A parental competence scale: Dimensions and their association with adolescent outcomes. Frontiers in Psychology.

[B42-behavsci-16-01623] Roman A., Catalano H., Barth K., Florescu C., Tipei-Voia M., Rad D., Chiș O., Demeter E., Roman R., Șandru R., Trifan I. M. (2026). Amotivation and academic engagement in Western Romanian university students: A conditional self-regulation model with forethought and self-reflection under perceived performance control. Brain Sciences.

[B43-behavsci-16-01623] Sagone E., De Caroli M. E. (2016). Are value priorities related to dispositional optimism and resilience? A correlational study. Contemporary Educational Researches Journal.

[B44-behavsci-16-01623] Sameroff A. J., Rosenblum K. L. (2006). Psychosocial constraints on the development of resilience. Annals of the New York Academy of Sciences.

[B45-behavsci-16-01623] Sandy C. J., Gosling S. D., Schwartz S. H., Koelkebeck T. (2017). The development and validation of brief and ultrabrief measures of values. Journal of Personality Assessment.

[B46-behavsci-16-01623] Schwartz S. H., Zanna M. P. (1992). Universals in the content and structure of values: Theoretical advances and empirical tests in 20 countries. Advances in experimental social psychology.

[B47-behavsci-16-01623] Skevington S. M., The WHOQOL SRPB Group (2020). Is culture important to the relationship between quality of life and resilience? Global implications for preparing communities for environmental and health disasters. Frontiers in Psychology.

[B48-behavsci-16-01623] Smith C., Carlson B. E. (1997). Stress, coping, and resilience in children and youth. Social Service Review.

[B49-behavsci-16-01623] Soare A.-C. (2025). Reziliența ca proces dinamic [Resilience as a dynamic process]. Valorificarea neuroștiințelor în dezvoltarea personală: Materialele Conferinței Științifice Internaționale, 7–8 noiembrie 2025.

[B50-behavsci-16-01623] Southwick S. M., Bonanno G. A., Masten A. S., Panter-Brick C., Yehuda R. (2014). Resilience definitions, theory, and challenges: Interdisciplinary perspectives. European Journal of Psychotraumatology.

[B51-behavsci-16-01623] Stern P. C., Dietz T., Guagnano G. A. (1998). A brief inventory of values. Educational and Psychological Measurement.

[B52-behavsci-16-01623] Tangelder Y. C., Johansen J. D., Abebe D. S. (2025). Resilience and mental health among children and youth with an immigrant background in Europe: A scoping review. Children and Youth Services Review.

[B53-behavsci-16-01623] Tok Y., Ünal M. (2020). Examining the correlation between resilience levels and math and science process skills of 5-year-old preschoolers. Research in Pedagogy.

[B54-behavsci-16-01623] Twum-Antwi A., Jefferies P., Ungar M. (2020). Promoting child and youth resilience by strengthening home and school environments: A literature review. International Journal of School & Educational Psychology.

[B55-behavsci-16-01623] Ungar M. (2008). Resilience across cultures. The British Journal of Social Work.

[B56-behavsci-16-01623] Ungar M. (2015). Practitioner review: Diagnosing childhood resilience—A systemic approach to the diagnosis of adaptation in adverse social and physical ecologies. Journal of Child Psychology and Psychiatry.

[B57-behavsci-16-01623] Ungar M., Connelly G., Liebenberg L., Theron L. (2019). How schools enhance the development of young people’s resilience. Social Indicators Research.

[B58-behavsci-16-01623] Ungar M., Liebenberg L. (2011). Assessing resilience across cultures using mixed methods: Construction of the Child and Youth Resilience Measure. Journal of Mixed Methods Research.

[B59-behavsci-16-01623] Ungar M., Liebenberg L., Boothroyd R., Kwong W. M., Lee T. Y., Leblanc J., Duque L., Makhnach A. (2008). The study of youth resilience across cultures: Lessons from a pilot study of measurement development. Research in Human Development.

[B60-behavsci-16-01623] Uzefovsky F., Döring A. K., Knafo-Noam A. (2016). Values in middle childhood: Social and genetic contributions. Social Development.

[B61-behavsci-16-01623] Vedder P., Berry J. W., Sabatier C., Sam D. L. (2009). The intergenerational transmission of values in national and immigrant families: The role of zeitgeist. Journal of Youth and Adolescence.

[B62-behavsci-16-01623] Voicu B., Voicu M. (2007). Valori ale românilor: 1993–2006. O perspectivă sociologică *[The values of Romanians: 1993–2006. A sociological perspective]*.

[B63-behavsci-16-01623] Werner E. E. (1989). High-risk children in young adulthood: A longitudinal study from birth to 32 years. American Journal of Orthopsychiatry.

[B64-behavsci-16-01623] Werner E. E. (1993). Risk, resilience, and recovery: Perspectives from the Kauai Longitudinal Study. Development and Psychopathology.

[B65-behavsci-16-01623] Yonker J. E., Schnabelrauch C. A., DeHaan L. G. (2012). The relationship between spirituality and religiosity on psychological outcomes in adolescents and emerging adults: A meta-analytic review. Journal of Adolescence.

[B66-behavsci-16-01623] Zolkoski S. M., Bullock L. M. (2012). Resilience in children and youth: A review. Children and Youth Services Review.

